# Gender Differences in Clinical and Psychosocial Features Among Persons With Schizophrenia: A Mini Review

**DOI:** 10.3389/fpsyt.2021.789179

**Published:** 2021-12-22

**Authors:** Giulia Maria Giordano, Paola Bucci, Armida Mucci, Pasquale Pezzella, Silvana Galderisi

**Affiliations:** Department of Psychiatry, University of Campania Luigi Vanvitelli, Naples, Italy

**Keywords:** schizophrenia, gender, course, remission, functional outcome

## Abstract

An extensive literature regarding gender differences relevant to several aspects of schizophrenia is nowadays available. It includes some robust findings as well as some inconsistencies. In the present review, we summarize the literature on gender differences in schizophrenia relevant to clinical and social outcome as well as their determinants, focusing on clinical variables, while gender differences on biological factors which may have an impact on the outcome of the disorder were not included herewith. Consistent findings include, in male with respect to female patients, an earlier age of illness onset limited to early- and middle-onset schizophrenia, a worse premorbid functioning, a greater severity of negative symptoms, a lower severity of affective symptoms and a higher rate of comorbid alcohol/substance abuse. Discrepant findings have been reported on gender differences in positive symptoms and in social and non-social cognition, as well as in functional outcome and rates of recovery. In fact, despite the overall finding of a more severe clinical picture in males, this does not seem to translate into a worse outcome. From the recent literature emerges that, although some findings on gender differences in schizophrenia are consistent, there are still aspects of clinical and functional outcome which need clarification by means of further studies taking into account several methodological issues.

## Introduction

During the last decades, several studies have explored differences between male and female patients with schizophrenia in several aspects of the disorder, including epidemiological distribution, clinical picture, course of illness, and biological correlates (1–4). The identification of these differences and the understanding of underlying biological and psychosocial mechanisms may contribute to clarifying the etiopathogenetic mechanisms of specific components of the disorder and to implement gender-tailored intervention strategies.

Proposed hypotheses to explain differences between male and female patients with schizophrenia include biological models (genetic, neurodevelopmental or hormonal), psychological aspects (different psychological vulnerability and/or trauma exposure between the two genders) and social factors (mainly related to cultural aspects such as different gender role expectations) (5–7).

Consistent findings have been reported so far for differences between males and females in the age of onset, premorbid functioning, negative and affective symptoms and substance use. In fact, the vast majority of relevant studies found, in male compared with female patients an earlier age of onset (probably limited to early- and middle-onset schizophrenia), a worse premorbid functioning, a greater severity of negative symptoms, a lower severity of affective symptoms and a higher frequency of alcohol/substance abuse (1–3). Differences between males and females in other aspects of the disorder, such as other psychopathological domains apart from negative and affective symptoms, neurocognition, social cognition and personal resources, have received scarce attention and/or relevant studies provided discrepant findings (2, 5, 8).

A less favorable illness outcome would be expected in male than in female patients with schizophrenia on the basis of main findings listed above, as the majority of factors showing gender differences, and reported as more severe in males, have a known impact on the outcome of schizophrenia (9–12). However, no conclusive findings have been reported as yet on gender differences in illness outcome.

In the light of the above observations, the present review is addressed to researchers interested in the study of these topics. It aims at providing an update on more recent major findings on differences between male and female patients with schizophrenia relevant to clinical and functional outcome. In addition, the review focuses on gender differences in clinical indices which may have an impact on outcome, namely, age of onset, premorbid functioning, psychopathology and social/non-social cognition, which may clarify interpretation of findings on outcome. It also aims at offering a contribution in highlighting questions still open and methodological factors which should be taken into account in further studies. Differences on biological aspects—such as genetic and neuromorphological findings—are not reported herewith.

We searched PubMed for articles in English published since 2016 including the following terms: (schizophrenia) AND (sex differences OR gender differences) AND (age of onset OR premorbid functioning OR symptoms OR cognition OR social functioning OR recovery). Other older reviews and landmark papers were selected from the reference list of the resulting articles. We included both terms “sex” and “gender” in our search. More often, the former is used to describe differences between males and females that are biologically determined, while the latter is defined as the result of socially constructed ideas about the behavior, actions and roles which a subject performs. However, given the strong interaction between biological and psychosocial aspects in influencing differences between males and females, not always a clear cut distinction is made. Here we report all findings by using the term “gender” in order to comprise the notion of psychosocial differences between males and females, given that the notion of biological differences is already included in the classification of patients according to the demographic variable male/female adopted in all examined papers.

## Age of Onset

An earlier age of illness onset has consistently been reported in male as compared to female persons with schizophrenia, with a difference ranging from 1 to 10 years. A peak of onset has been described in males in their early- to mid-twenties, while in females it has been reported in their late-twenties. Many authors also reported a second smaller peak of onset in females after age 40 (1, 2, 5, 13–19). Only a few studies have reported a lack of gender differences in age of onset (20, 21). According to the findings of a meta-analysis (22) and of a more recent paper (23), gender differences in age of onset are not confirmed in patients with a family history of psychosis, suggesting that the genetic loading for schizophrenia may overcome the effects of protective factors in females, thus reducing their age of onset and making it closer to that of males (22).

Among possible explanations of later age of onset in females, one of the most frequently hypothesized is the protective role of estrogens from excessive dopamine turnover, which would also explain the second incidence peak around menopausal age in women (2, 13), as well as the frequent onset of psychosis in the immediate post-partum (24). Other possible explanations have been proposed, which do not exclude the hormonal hypothesis, but may have a complex relationship with it. One is that of genetic polymorphism within the transforming growth factor-β, which influences early development of neuronal system, with the CC genotype that was found to be associated to later age of onset only in females (25). Another explanation would be the worse premorbid functioning reported in males, which could have an impact on the age of onset (23), however, this association may also be interpreted in the opposite direction, i.e., the worse functioning reported in males before the psychosis onset may be due to the presence of prodromal symptoms (see below) which, in turn, worsen functioning at an early age. A higher frequency of cannabis use in males could be a further possible cause of earlier illness onset, although studies controlling for this variable did not support this hypothesis (2). It has been questioned whether the finding of a later age of onset in females can be an artifact related to delayed diagnosis in females, possibly related to the presence of affective symptoms leading to a higher probability of misdiagnosis in females than in males (16), or to the delay in help seeking that has been reported, although non-consistently, in females (2).

## Premorbid Functioning

A worse premorbid adjustment has been reported in male vs. female patients in several studies carried out in patients with chronic schizophrenia or with first-episode psychosis (1, 2, 26, 27). Although this is a consistent finding, it is not clear whether the disadvantage reported in males is limited to a specific domain of functioning. In fact, premorbid functioning is not a unitary construct, but includes at least two distinct domains, namely the academic or the social one (28) which may have different relationships with psychopathology, cognition and functional outcome (29–31).

Studies on gender differences exploring separately social and academic domains reported discrepant findings, as a worse premorbid functioning has been found in males only for the social domain (32–34), or only for the academic one—as reported in three studies carried out in large samples of patients using state-of-the art tools to assess premorbid functioning by directly interviewing the patient and/or a family member (27, 35, 36). One study carried out in a small sample of patients reported a worse premorbid functioning in males for both domains (37). Discrepancies may be related, at least in part, to the retrospective nature of the assessment of premorbid functioning, which might suffer from recall biases. Another issue in the assessment of premorbid functioning is the risk of inclusion of prodromal symptoms as characteristics of premorbid functioning. It has been questioned whether the worse functioning reported in males before the development of psychosis is really premorbid or rather related to the presence of prodromal symptoms, given the earlier age of onset reported in males (2, 26). Although this hypothesis cannot be ruled out, it must be noticed that in some studies a worse functioning in males is reported during childhood and early adolescence (27, 35), i.e., during life periods in which, with respect to late adolescence and adulthood, the risk of contamination of premorbid assessment with early prodromal symptoms is reduced. Moreover, the hypotheses that schizophrenia may be viewed within a “developmental risk factor model” (38) or within a “neurodevelopmental continuum” (39), the latter more frequent in males, support the possibility of a premorbid impairment at an early age.

## Psychopathological Features

Studies exploring gender differences with respect to psychopathological features of schizophrenia consistently reported a higher frequency of affective symptoms in females vs. males, both in chronic patients (2, 5, 40–42) and in patients with first-episode psychosis (2, 5, 26, 43). In females, a higher frequency of comorbid affective disorders has also been reported in some studies (17, 44), as well as a higher rate of suicide attempts (17, 45) and a lower rate of completed suicide (46–49). The potential explanations of these findings are unclear. In females, the higher frequency of past history of sexual abuse (50, 51) may be one of the possible explanations of the higher levels of depressive symptoms and disorders, given the association reported between sexual abuse and depressive symptoms (50) as well as the mediating effect of depressive symptoms in the association between childhood sexual abuse and psychosis, although the latter has been found limited to less severe forms of sexual abuse (52). Another hypothesis is that, given the frequently reported association of depressive symptoms in schizophrenia with illness insight (53, 54), low mood in females may be at least in part a consequence of their greater illness insight (55). However, the finding of a better insight in females reported by some authors (56, 57) has not been confirmed by a number of studies (58–62), and the concept itself of insight in schizophrenia remains controversial (63). The finding of more suicide attempts in females is indeed expected on the basis of their higher rate of depressive symptoms and comorbidity for depression, while the higher frequency of suicide in males may be explained by clinical and psychosocial factors such as their greater access to firearms, their more severe social withdrawal and isolation, and their aggressive urges that can be turned toward the self (3).

Another frequently reported gender difference in psychopathology is a greater severity of negative symptoms in males vs. females (1, 2, 5, 13, 15, 16, 18, 40, 64–68). This finding has been confirmed in patients with first-episode psychosis (43, 69, 70), as well as in older adults with schizophrenia (65, 71). In the majority of studies, the finding is referred to negative symptoms measured with outdated instruments and often considered as a unitary construct, while the most recent literature suggests that these symptoms are heterogeneous and include at least two factors—“avolition” and “poor emotional expression”—that might be underpinned by different pathophysiological substrates and show different correlates (72). The only study in which the two factors were separately analyzed (71) found that the greater severity of negative symptoms in males was limited to the experiential deficit. Another study which analyzed scores of subscales assessed with a new generation rating scale for negative symptoms (68) found that males had a significantly higher score only on the total as well as on the subscales “distress” and “asociality.” No study took into account the distinction between primary and secondary negative symptoms.

Findings relevant to the psychopathological positive dimension are inconsistent. A higher frequency of positive symptoms was reported in females than in males in several papers (5, 42, 67, 69, 73, 74), including a study carried out in a large sample of patients with first-episode schizophrenia (69), while others reported an opposite pattern of differences (2). Interestingly, in a study exploring gender differences across diverse regions of the world (64), a greater severity of positive symptoms in females was found only in some regions, suggesting that psychosocial and cultural factors can impact the severity of this psychopathological dimension with a differential effect on gender. In addition, such regional differences may represent one possible cause of discrepancies among studies, along with differences in the scales adopted to assess positive symptoms, the criteria defining the positive domain or the medications assumed by the patients.

A few studies investigated disorganization, and reported discrepant data with either no gender differences (42, 43) or greater severity in males (75).

A lack of gender differences on psychopathological domains has also been reported in some studies (1, 26, 76), while others have found gender differences with respect to psychopathology only in patients with late-onset (over 40 years) schizophrenia (2, 16). This latter finding is in line with the hypothesis of an attenuation of gender differences over time (see below).

## Impairment in Neurocognition and Social Cognition

Cognitive deficits are considered central and persistent features of schizophrenia and have a significant impact on outcome and quality of life (9, 77–79).

The characterization of gender differences in neurocognitive profiles of people with schizophrenia, and the study of their possible explanations, may contribute to the progress of knowledge on the pathogenetic mechanisms underlying cognitive deficit in schizophrenia and on their impact on functional outcome. Unfortunately, findings on this topic are still controversial. The review by Mendrek and Mancini-Marïe (8) highlighted that several studies found better neurocognitive functions in female than in male patients, while others found an opposite pattern or no gender difference. Findings of more recent studies did not clarify this picture, as a greater neurocognitive impairment has been reported in males (15, 71, 80–82) but also in females (83), or no gender differences (67). Moreover, when gender differences were found, the patterns of impaired cognitive domains in males and females varied among studies. According to some authors, cognitive domains more frequently impaired in males include attention, immediate and delayed memory, and executive functions, while in women they involve visuospatial and attention indices as well as verbal and spatial memory (8, 15, 80, 84). However, different neurocognitive profiles have been described in males and females in other studies, such as a greater impairment in males on reasoning and problem solving, processing speed and working memory (80). Studies investigating gender differences in changes of neurocognitive impairment over time also reported discrepant findings with lack of differences (85) or more stable deficits in males (86). Discrepancies in these findings may be due to the heterogeneity of clinical expression in schizophrenia (8), such as the presence/absence of severe negative symptoms in the clinical picture, as they have been found correlated to cognitive impairment in several studies (79, 87). Also methodological factors such as differences in sample size or the heterogeneity of tests used to assess cognitive functions may account for discrepancies among studies; as a matter of fact, among the above reported studies, only three (80, 82, 83) assessed neurocognitive domains by means of the MATRICS Consensus Cognitive Battery, which is considered the gold standard to reliably assess neurocognitive functions in subjects with schizophrenia (88).

A few studies explored gender differences in social cognition, a domain relatively independent of neurocognition, although related to it. Social cognition is the subject's ability to perceive, interpret and process social stimuli for adaptive social interactions (89–91). Several studies found that it is associated to functional outcome even more strongly than neurocognition, acting as mediator between the latter and functional outcome (9, 92–96).

Although non-conclusive data have been reported so far on this topic, a disadvantage in males vs. females has been found in some studies with respect to social cognition (66, 80, 84, 97). However, discrepant findings have also been reported, as no gender differences in patients with schizophrenia have been found (68, 98, 99).

## Outcome

The concept of outcome in schizophrenia, initially regarded as reduction of relapses and symptom severity, then as symptomatic remission, nowadays includes prevention and treatment of comorbidities, as well as the improvement in functional outcome and the subjective well-being which are considered important targets of the care of people with schizophrenia (100).

The indices of outcome considered in the literature on gender differences in schizophrenia are quite heterogeneous. They include socio-demographic variables, such as marital status and employment, which may indirectly represent—although not necessarily—indices of a favorable illness outcome; clinical indices such as number and duration of hospitalizations; behavioral characteristics, physical comorbidities and symptomatic remission; response to antipsychotic drugs; social functioning and quality of life.

### Socio-Demographic and Clinical Indices of Outcome

A lower frequency of being partnered/married has been reported in males in most (1, 5, 15, 101, 102) although not all (55, 103) studies. It has been hypothesized that this is related to the preponderance of negative symptoms in males' clinical picture or to their earlier illness onset, which may both have stood as barriers to develop intimate relationships. However, as argued by Seeman (3), being married may not necessarily represent a good index of outcome, as it depends on the quality of the partner relationship.

Higher rates of comorbid alcohol/substance abuse have been consistently reported in males (2, 14, 20, 27, 104, 105). However, this seems to be an aspecific index of outcome, since it has been reported in males also in the general population (106); also, it may be considered as a risk factor rather than an index of functioning. As a matter of fact, although it is associated with several social and behavioral unfavorable indices, such as violence, unemployment or homelessness, its relationship with poor functional outcome has not been confirmed (3).

Longer hospital stays and higher frequency of readmissions (1, 3, 55, 101) have been reported in males. However, there are also reports of higher rates of admissions in females (103).

A further index of outcome deserving attention in patients with schizophrenia is the presence of physical comorbidities that have a significant impact on functional outcome (107) and represent 60% of causes of excess mortality reported in this population of patients (108, 109). Studies investigating gender differences with respect to life expectancy in patients with schizophrenia reported either a lack of difference in the mortality ratio of patients with schizophrenia vs. the general population (110–112) or a greater reduction of life expectancy with respect to the general population in male vs. female patients with schizophrenia (113–115). A higher frequency of cardiovascular death has been found in male (3, 17), but also in female patients (116) as well as a higher frequency of death caused by cancer in females (17). An association between antipsychotic use and reduced all-cause, cardiovascular and suicide mortality has been reported in both genders (117).

### Functional Outcome and Recovery

Gender differences in the rate of employment have been explored among indices of functional outcome. Some studies reported a lower rate of employment in male patients (55, 118), however, this finding was not confirmed in other studies reporting an opposite pattern of differences between males and females (119) or no gender difference in the rate of employment (66, 120). This heterogeneity of findings may be due at least in part to the great impact of social and cultural factors on this variable, as confirmed in the study on regional differences in schizophrenia outcome by Novick et al. (64), which reported a higher rate of employment in males in East Asia and North Africa, but not in other regions of the world.

Studies on gender differences relevant to other indices of functional outcome (i.e., social functioning and quality of life) also produced inconsistent findings so far. The review by Ochoa et al. (1) provides a detailed report on this regard, highlighting that many studies found an advantage in female patients with chronic or first-episode schizophrenia on social functioning while two studies in chronic patients found no gender differences (75, 121)These inconsistencies have not been clarified by subsequent papers. A study with a prospective design with 5-year follow-up carried out in the large sample of patients (118), found a better social functioning in females. In more recent papers, a better social functioning in females has been confirmed (14, 68, 86, 122), however, also a lack of gender differences has been reported (123). In a study assessing several areas of social functioning, the advantage of females was limited to areas related to independence and competence in daily life (124).

A similar picture arises from studies investigating gender differences in quality of life, as both a lack of gender differences (19, 120, 125) or an advantage of females in some domains of quality of life (66) have been reported.

As to recovery, a large meta-analysis (126) reported no gender difference in rates of recovery defined as improvement in both clinical and social domain persisting for at least 2 years. Lack of gender differences in the recovery rates has been confirmed in other studies (75, 123) but a higher rate of recovery in females has also been found (118).

The impact of social and cultural factors on gender differences in functional outcome has been highlighted by findings of Novick et al. (64) who reported a higher frequency of functional remission in females (based on rates of employment, independent living and active social interaction) only in some regions of the world. As a matter of fact, gender differences on potential social disadvantages related to the cultural background have been reported in people with schizophrenia. They include higher frequency of sexual abuse, socioeconomic disadvantage and increased responsibility for the care of others in females, lower family toleration for symptomatic behaviors in males (6).

Moreover, some studies found that gender differences in outcome indices and rates of recovery depend on the time of evaluation, with better outcome for women in the short- and mid-term (up to 10 years of illness duration) and an attenuation of gender differences in the long-term (13–40 years), as well as on the age of onset, with females showing a better course when the onset is up to age 40 and a worse outcome when the onset occurs later (3, 127). In a recent paper, Dama et al. (27) reported that females show a better social functioning after 1 year of treatment, but not after 2 years, whilst higher rates of clinical remission in females were confirmed after 1 and 2 years of treatment. These findings suggest two possible explanations: that males require more than 1 year to achieve a good functioning, or that female advantage is limited to the initial stages of illness. In line with the latter hypothesis, Kohler et al. (128) found better functional outcome in females than males, but this pattern was inverted in patients at very late onset of illness (over 65 years). The reduction of estrogen levels with the menopause might account for the attenuation of the advantage in the course of illness at later stages in females.

## Discussion

The literature addressing gender differences in people with schizophrenia shows that males, as compared to females, have an earlier age of illness onset—limited to early- and middle-onset schizophrenia—a worse premorbid functioning, a greater severity of negative symptoms, a lower severity of affective symptoms and a higher rate of comorbid alcohol/substance abuse. Despite the overall finding of a more severe clinical picture in males, this does not seem to translate into a worse outcome. In fact, inconsistent findings have been provided so far on gender differences in illness outcome.

This may be due to several reasons, as detailed below.

First, although some aspects of gender differences have already been confirmed in many studies, they may still need further investigation. As an example, it would be useful to explore whether the greater severity of negative symptoms, widely reported in male patients, involves all or only some domains of this complex and heterogeneous psychopathological dimension, since it has been found that different domains of negative symptoms—namely “avolition” and “poor emotion expression”—may have a differential impact on outcome (72, 129). Therefore, it is important that further studies use state-of-the-art instruments to assess complex domains such as negative symptoms and cognitive functions.

Second, some domains of schizophrenia for which findings on gender differences are still inconclusive (e.g., neurocognition, social cognition and physical comorbidities) are important determinants of social functioning (9, 95, 96, 107, 130–132). Therefore, reliable data on gender differences in these factors may shed light on gender differences in outcome.

Third, the complexity related to the concept of outcome in schizophrenia is probably among the main sources of discrepancies between studies investigating illness outcome in male and female patients with schizophrenia. In fact, outcome in schizophrenia was initially regarded as the improvement of symptom severity and reduction of the number of relapses, then its definition moved to symptomatic remission, and more recently to good functional outcome and recovery (100, 133). Good functional outcome includes the ability to function in the community, socially and vocationally, and the perception of a good quality of life (100) and is nowadays recognized as the main goal of schizophrenia treatment, given the lack of functional recovery observed in most affected people, despite important progress in the treatment of schizophrenia (134, 135). Moreover, recovery is a multifaceted construct including subjective/personal recovery on one hand, and objective/clinical recovery on the other hand. Personal recovery, refers to the subjective experience of recovery, defined by the quality of life, hope, reliance on others and not feeling overwhelmed by symptoms (136). Therefore, inconsistencies in gender differences in outcome may be related to the heterogeneous definition of indices of outcome among studies, to the complexity of the concept of functional outcome which includes different areas of real-life functioning (i.e., community activities, interpersonal relationships, work) that may be differentially impaired and/or may differentially improve after treatment in the two genders, as well as to the lack of differentiation between personal and clinical recovery which may be influenced by different factors.

Fourth, social and cultural background of the study population, which has been reported to influence gender differences on functional outcome (6), not always has been taken into account in the research on this topic.

Fifth, several studies did not take into account age of onset and duration of illness of the study population which should always be controlled for, given the fact that several authors reported an attenuation of gender differences in the illness outcome in patients with late-onset schizophrenia as well as in the later stages of illness.

Sixth, other variables which have a possible direct or indirect impact on outcome and may differ between genders, such as personal resources—which may also be in relationship with negative symptoms—and stigma (9, 79) or physical comorbidity (17), have been scarcely investigated as yet, therefore their inclusion in further studies is advisable.

On the whole, the above observations suggest that further studies are needed to clarify gender differences in clinical and functional outcome of schizophrenia, which should take into account the suggested methodological issues. The improvement of knowledge in this field could contribute to clarify etiopathogenetic mechanisms of specific components of the disorder, identify the factors influencing functional outcome and develop gender-tailored intervention strategies.

## Author Contributions

GG and PP performed the literature search. GG and PB drafted the article. AM and SG critically revised the work. All authors contributed to the article and approved the submitted version.

## Conflict of Interest

SG has been consultant and/or advisor to and/or received honoraria or grants from Innova Pharma-Recordati Group, Janssen Pharmaceutica NV, Sunovion Pharmaceuticals, Gedeon Richter-Recordati, Lundbeck and Angelini. The remaining authors declare that the research was conducted in the absence of any commercial or financial relationships that could be construed as a potential conflict of interest.

## Publisher's Note

All claims expressed in this article are solely those of the authors and do not necessarily represent those of their affiliated organizations, or those of the publisher, the editors and the reviewers. Any product that may be evaluated in this article, or claim that may be made by its manufacturer, is not guaranteed or endorsed by the publisher.
